# β3-adrenoceptor agonist prevents alterations of muscle diacylglycerol and adipose tissue phospholipids induced by a cafeteria diet

**DOI:** 10.1186/1743-7075-1-4

**Published:** 2004-08-17

**Authors:** Christian Darimont, Marco Turini, Micheline Epitaux, Irène Zbinden, Myriam Richelle, Eulàlia Montell, Andreu Ferrer-Martinez, Katherine Macé

**Affiliations:** 1 Nestlé Research Center, P.O. Box 44, Vers-Chez-Les-Blanc, 1000 Lausanne 26, Switzerland; 2 Department de Bioquimica i Biologia Molecular, Universitat de Barcelona, Barcelona, Spain

## Abstract

**Background:**

Insulin resistance induced by a high fat diet has been associated with alterations in lipid content and composition in skeletal muscle and adipose tissue. Administration of β3-adrenoceptor (β3-AR) agonists was recently reported to prevent insulin resistance induced by a high fat diet, such as the cafeteria diet. The objective of the present study was to determine whether a selective β3-AR agonist (ZD7114) could prevent alterations of the lipid profile of skeletal muscle and adipose tissue lipids induced by a cafeteria diet.

**Methods:**

Male Sprague-Dawley rats fed a cafeteria diet were treated orally with either the β3-AR agonist ZD7114 (1 mg/kg per day) or the vehicle for 60 days. Rats fed a chow diet were used as a reference group. In addition to the determination of body weight and insulin plasma level, lipid content and fatty acid composition in gastronemius and in epididymal adipose tissue were measured by gas-liquid chromatography, at the end of the study.

**Results:**

In addition to higher body weights and plasma insulin concentrations, rats fed a cafeteria diet had greater triacylglycerol (TAG) and diacylglycerol (DAG) accumulation in skeletal muscle, contrary to animals fed a chow diet. As expected, ZD7114 treatment prevented the excessive weight gain and hyperinsulinemia induced by the cafeteria diet. Furthermore, in ZD7114 treated rats, intramyocellular DAG levels were lower and the proportion of polyunsaturated fatty acids, particularly arachidonic acid, in adipose tissue phospholipids was higher than in animals fed a cafeteria diet.

**Conclusions:**

These results show that activation of the β3-AR was able to prevent lipid alterations in muscle and adipose tissue associated with insulin resistance induced by the cafeteria diet. These changes in intramyocellular DAG levels and adipose tissue PL composition may contribute to the improved insulin sensitivity associated with β3-AR activation.

## Background

Dietary fatty acids are known to influence the composition of stored triacylglycerol (TAG) and membrane phospholipids (PL) in adipose tissue [1]. More recently, it was demonstrated that the lipid profile in skeletal muscle reflected dietary lipids [2-4]. Furthermore, the modifications of fatty acid concentrations and composition in tissue lipids induced by a high fat diet has been associated with alterations in lipid metabolism and insulin sensitivity [5,6]. Indeed, enrichment of membrane PL with saturated fatty acids (SFA) was able to impair insulin action in skeletal muscle and adipose tissue, whereas a higher proportion of polyunsaturated fatty acids (PUFA) improved insulin sensitivity in these tissues [7-9]. TAG accumulation in skeletal muscle was also correlated with the development of insulin resistance, independent to the degree of obesity [10-13]. Intramyocellular TAG could represent only a marker of insulin resistance whereas intracellular accumulation of long chain acyl-coenzyme A, ceramide or diacylglycerol (DAG) were reported to directly alter the insulin action [14].

Chronic activation of the β3-adrenoceptor (β3-AR), which is predominantly expressed in white and brown adipose tissue, by selective agonists exerts both anti-obesity and anti-diabetic effects in rodent models of obesity [15,16]. Activation of this receptor has been reported to enhance energy expenditure via stimulation of thermogenesis in brown adipose tissue [16]. The improvement in glucose homeostasis induced by β3-AR agonists appears to be a consequence of increased insulin sensitivity in peripheral tissues rather than stimulation of insulin secretion by the pancreas [17]. Although, the expression of β3-AR in myocytes is still a matter of debate [18-20], obese rats treated with β3-AR agonists demonstrated an improvement of insulin sensitivity in brown and white adipose tissue as well as in skeletal muscle [17,21,22]. In adipose tissue this effect is believed to be mediated by the conversion of large adipocytes into small adipocytes, which are more sensitive to insulin [21]. In skeletal muscle, it seems more likely that the effects of β3-AR agonists on insulin sensitivity are mediated by alternate indirect mechanisms.

The objective of the present study was to determine whether a selective β3-AR agonist could prevent alterations in the profile of skeletal muscle and adipose tissue lipids induced with the consumption of the cafeteria diet, previously reported to induce weight gain and hyperinsulinemia [22,23]. The selective β3-AR agonist ZD7114 was used in this study. When administered at 1 mg/kg/day, this compound has been shown to increase thermogenesis in rodents and dogs without increasing heart rate or β2-AR-mediated effects such as tremor [24]. ZD7114 pharmacological specificity was also demonstrated in brown adipocytes and smooth muscles [25,26]. As expected, we observed that a chronic treatment with the ZD7114 prevented the development of excessive fat mass and hyperinsulinemia induced by this diet. These preventive beneficial effects exerted by the β3-AR agonist were associated with a reduction of muscle DAG accumulation. In adipose tissue, ZD7114 treatment was able to limit the proportional reduction of PUFA into the PL pool, induced by the cafeteria diet. Our results indicate that a β3-AR agonist prevents some cafeteria diet-induced alterations of the fatty acid profile of lipids in skeletal muscle and adipose tissue. Furthermore, we propose that these changes may contribute to the improved of insulin sensitivity observed in rats treated with a β3-AR agonist during the development of obesity.

## Methods

### Animal study

Male Sprague-Dawley rats were purchased from Charles River (L'arbesles, France) at 5–6 weeks of age. Animals were individually housed in temperature-controlled rooms (22°C) with a 12-h light-dark cycle. Ten days before the beginning of the study, 30 rats were provided a normal chow diet (Kliba-Nafalg, Switzerland) and free access to drinking water. At the end of this period, rats were weighed and pre-selected for their sensitivity to weight gain (i.e. rat presenting at least 35% weight gain after a 10-day selection period). The selected rats (n = 15) were equally randomized into 3 groups. A reference group, consisted of rats fed for 60 days with a standard pellet chow diet containing 28, 57 and 15% E from protein, carbohydrate and fat, respectively (REF group). The remaining rats were fed for 60 days with a cafeteria diet composed of 30 g of a mix containing salami, cookies, cheese, sausage, chips, chocolate and almonds in a proportion of 2:2:2:1:1:1:1 and 30 g of the reference group chow diet. This mixed diet contained 26, 27 and 47% energy as protein, carbohydrate and fat, respectively. At the beginning of the dietary intervention, both groups fed the cafeteria diet received daily, by gavage (0.5 ml/100 g body weight), either the selective β-3AR agonist ZD7114 at 1 mg/kg per day (CAF-ZD group) or water alone (CAF group) until day 60. Rats from the REF group (chow diet) also received a daily gavage of water (0.5 ml/100 g body weight).

Body weight and food intake were recorded daily. Rats were fasted for 8 hours before sacrifice, performed under isoflurane anaesthesia. Tissues were immediately collected, weighed, frozen in liquid nitrogen and kept at -80°C until analysed.

All procedures in the study were in compliance with the ethical committee of the "Service vétérinaire du canton de Vaud".

Estimation of the proportions of the different lipid classes contained in the cafeteria diet (see Discussion) was performed using the USDA Nutrient Database.

### Lipid fatty acid composition

#### Adipose tissue

Fatty acid composition of lipids in adipose tissue was performed by Lipomics Technologies (West Sacramento, USA). Briefly, lipids were extracted from 30 mg of frozen epididymal adipose tissue in the presence of authentic internal standards by the method of Folch et al. [27] using chloroform:methanol (2:1, by vol.). After separation of individual lipid classes by preparative thin-layer chromatography, the TAG and PL were scraped and trans-esterified; the resulting fatty acid methyl esters were then separated and quantified by capillary gas chromatography as previously described [28].

#### Muscle

The frozen gastrocnemius muscle was thawed and thoroughly dissected under stereomicroscope to remove extramyocellular adipose tissue. Lipids from lyophilized, finely powdered, dissected muscle (50 mg) were extracted according to the method of Folch [27]. PL (19:0), TAG (17:0) and DAG (15:0) internal standards (Varian; Zug, Switzerland) were added prior to lipid extraction. The lipids were loaded on a Chromabond NH2 cartridge (Varian; Zug, Switzerland), and neutral lipids were separated from free fatty acids and PL by sequential elution with chloroform/2-Propanol (2:1), 2% acetic acid in diethylether and methanol, respectively [29]. The neutral lipids were subjected to thin-layer chromatography using hexane:diethylether:acetic acid (70:30:1, by vol.) as solvent system to separate DAG from TAG [30]. The hydrolysis of TAG into DAG during sample analysis has been assessed and represented less than 0.5% of total DAG. The fatty acids from the phospholipids, TAG and DAG were converted to their methyl esters. Fatty acid methyl-ester separation was performed by automated gas-liquid chromatography (HP 6890 series) with FID detection (280°C); authentic standard mixtures of fatty acid methyl-esters (Nu-Chek-Perp; Lowell Mutter, USA) were injected to identify fatty acid methyl-ester peaks. Results are expressed in μmol fatty acids per gram lyophilised muscle.

### Plasma metabolites

Plasma glucose and insulin were determined with commercially available kits purchased from Sigma (Buchs, Switzerland) and Crystal Chem Inc. (Downers Grove, USA), respectively. Plasma triglycerides and free fatty acids concentrations were analysed using kits from Roche Diagnostic (Basel, Switzerland) and Wako Chemicals (Richmond, USA), respectively.

### Statistical analysis

Comparisons of the means of the dependent variables of each group were performed using a one-way ANOVA.

## Results

### Body weight and fat mass

Male Sprague-Dawley rats were fed, during 60 days, either a chow diet used as a reference group (REF group), a cafeteria diet (CAF rats) or a cafeteria diet plus a daily gavage of the β3-AR agonist ZD7114 (CAF-ZD rats).

**Figure 1:**
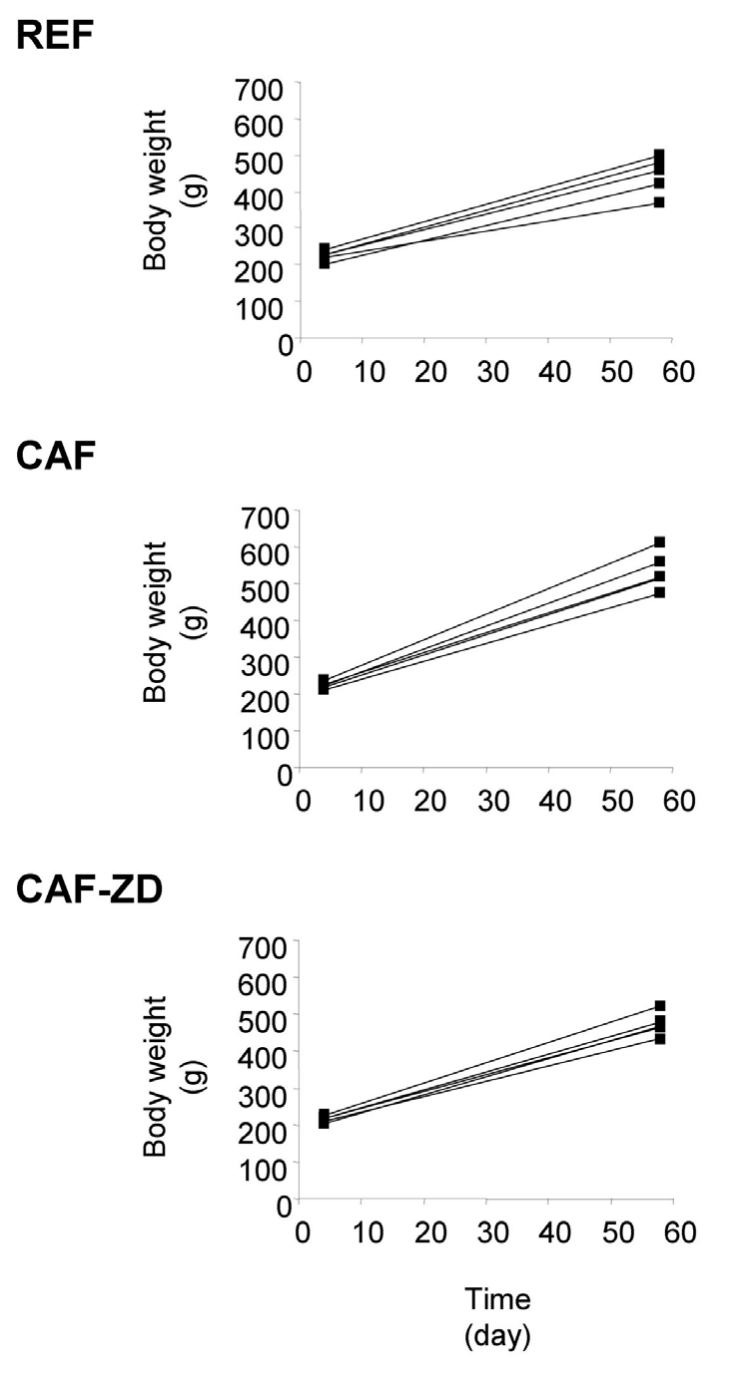
Individual weight changes. Body weight was measured in rats (n = 5) fed a chow diet (REF) or a cafeteria diet alone (CAF) or treated with 1 mg/kg/day ZD7114 (CAF-ZD) at the beginning and at the end of the different interventions (day 60). Data are represented as individual values.

At day 60, the mean body weight of CAF rats was significantly greater than that of REF rats (Table 1). On the other hand, CAF-ZD rats presented a significant reduction in mean body weight when compared with CAF rats (Table 1). Similar effects were observed on weight gain which was 46% greater in CAF rats than in REF rats (Figure 1; 213.2 ± 12.2 g vs. 312.3 ± 19.2 g in REF and CAF rats, respectively; p < 0.01), and reduced by 17% in CAF-ZD rats as compared to CAF rats (258.70 ± 12.70 g in CAF-ZD rats, p < 0.05). The enhancement of body weight in CAF rats was strongly associated with the weight increase of two main deep adipose depots, confirming the obesigenic properties of the cafeteria diet. Indeed, the epididymal and retroperitoneal fat pads in CAF rats were respectively 131% and 185% heavier than those of REF rats (Table 1). ZD7114 treatment decreased the weight of the two adipose tissue depots by about 45%, compared to CAF rats (Table 1). The weights of gastrocnemius skeletal muscle, liver and heart were not different between groups (Table 1). As expected, CAF rats had a higher energy intake compared with REF animals (106.03 ± 7.25 vs 78.49 ± 2.96 kcal/day). However, the anti-obesity effect of ZD7114 was not attributed to a reduction of energy intake (107.70 ± 7.30 kcal/day in CAF-ZD rats).

**Table 1:** Body weight, tissues and fasting plasma metabolites

	**REF**	**CAF**	**CAF-ZD**
**Final body weight (g)**	443.78 ± 21.42	540.20 ± 16.69 *	477.82 ± 12.09 +
**Retroperitoneal (g)**	3.73 ± 0.44	10.65 ± 0.48 **	5.78 ± 0.23 +
**Epididymal (g)**	4.65 ± 0.41	10.73 ± 0.43 **	5.96 ± 0.30 +
**Gastrocnemius (g)**	2.69 ± 0.13	2.78 ± 0.01	2.79 ± 0.03
**Heart (g)**	1.25 ± 0.07	1.48 ± 0.01	1.46 ± 0.02
**Liver (g)**	13.53 ± 0.79	16.69 ± 0.15	15.47 ± 0.12
**Glucose (mmol/l)**	12.80 ± 1.51	12.10 ± 1.02	14.78 ± 1.11
**Insulin (μU/ml)**	19.75 ± 2.05	98.04 ± 16.01 **	43.27 ± 8.26 +
**Fatty acid (mmol/l)**	0.47 ± 0.22	0.42 ± 0.68	0.30 ± 0.39 **
**Triacylglycerol (mmol/l)**	2.27 ± 0.30	4.59 ± 0.58 **	4.11 ± 0.95

Data are the mean ± SEM. Values significantly different from those obtained in the group of rats fed a chow diet (REF) are indicated by * (p < 0.05) and ** (p < 0.01). Data measured in the group of rats treated with ZD7114 (CAF-ZD) and significantly different from those in the group of rats fed a cafeteria diet (CAF) are shown by + (p < 0.05).

### Glucose and insulin plasma concentrations

Measurement of insulin and glucose concentrations in plasma of fasted animals showed that CAF rats presented a marked hyperinsulinemia with a 4.6 fold increase in insulin concentration as compared to REF animals whereas the glucose level was not changed (Table 1). ZD7114 treatment limited the hyperinsulinemia induced by the cafeteria diet, as demonstrated by the 2.3 fold decrease in plasma insulin concentrations in CAF-ZD rats compared with the CAF group (Table 1). No significant changes in plasma glucose concentrations were observed between these two groups (Table 1).

### Adipose tissue lipids

#### Lipid content

PL and TAG contents per gram of tissue were measured in epididymal adipose tissue of rats fed with a chow diet or a cafeteria diet treated or not with ZD7114. No significant change was observed in the concentration of PL and TAG in adipose tissue of CAF rats when compared with REF animals (Table 2). Furthermore, ZD7114 treatment did not affect adipose tissue lipid content of CAF rats.

**Table 2:** Adipose tissue lipid content

μmole/g tissue	**REF**	**CAF**	**CAF-ZD**
**Phospholipids**	8.29 ± 1.28	10.59 ± 1.06	8.65 ± 0.87
**Triacylglycerol**	2476.34 ± 68.31	2462.63 ± 41.33	2271.57 ± 122.46

Data are the mean ± SEM. REF: rats fed a chow diet; CAF: rats fed a cafeteria die, CAF-ZD: rats fed a cafeteria diet and treated with ZD7114

#### Fatty acid composition

**Figure 2:**
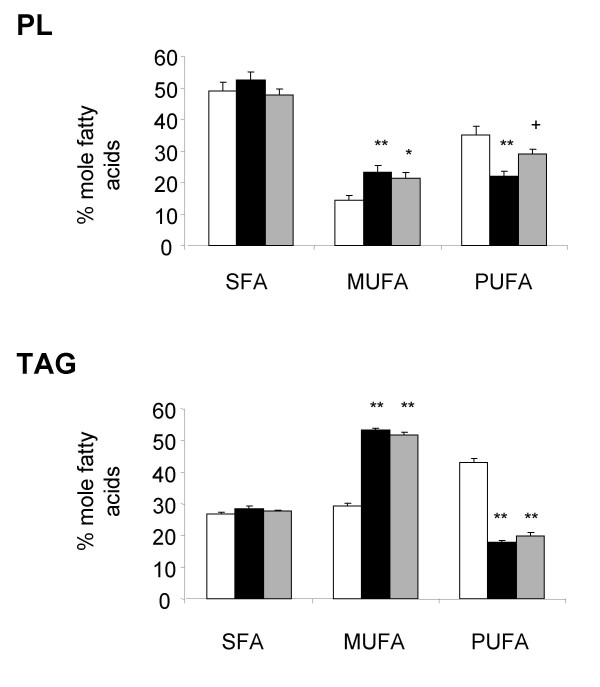
Proportion of the different lipid classes in adipose tissue. Saturated (SFA), monounsaturated (MUFA) and polyunsaturated fatty acids (PUFA) were measured in epididymal adipose tissue phospholipid (PL) and triacylglycerol (TAG) of rats fed a chow diet (empty bars) or a cafeteria diet (black bars) alone or treated with ZD7114 (grey bars). Data are represented as mean ± SEM, and values significantly different to data measured in rats fed the chow or cafeteria diets are indicated by * (p < 0.05), ** (p < 0.01) or + (p < 0.05), respectively.

Analysis of the fatty acid profile shows that cafeteria diet induced an increase in the proportion of monounsaturated fatty acids (MUFA), which was compensated for by a reduction in the percentage of PUFA in both adipose PL and TAG (Figure 2). Changes in the proportion of MUFA in PL and TAG were mainly due to the increase of oleic acid (1.7 and 2.0 fold increase in PL and TAG, respectively; Table 3). Reduction in the percentage of linoleic acid was mainly responsible for the decrease in proportion of PUFA in both PL and TAG (1.6 and 2.3 fold decrease for PL and TAG, respectively; Table 3). The proportions of arachidonic and α-linolenic acids were also slightly decreased in PL and TAG, respectively (Table 3). Although the global proportion of SFA was not modified in adipose tissue PL and TAG of CAF rats when compared with REF animals, the percentage of myristic and stearic acids were respectively enhanced in PL and TAG of CAF animals.

**Table 3:** Composition of adipose tissue lipids

% mole fatty acids	**REF**	**CAF**	**CAF-ZD**
**Phospholipids**			
Myristic acid (14:0)	3.04 ± 0.16	3.81 ± 0.19 *	2.96 ± 0.15 ++
Palmitic acid (16:0)	28.70 ± 3.10	30.72 ± 1.83	26.22 ± 1.70
Stearic acid (18:0)	15.99 ± 1.19	16.10 ± 0.75	16.93 ± 0.50
Palmitoleic acid (16:1n-7)	1.17 ± 0.28	1.35 ± 0.31	1.16 ± 0.29
Oleic acid (18:1n-9)	10.74 ± 0.94	18.17 ± 1.60 **	16.26 ± 1.39 *
Vaccenic acid (18:1n-7)	1.24 ± 0.12	0.89 ± 0.07 *	0.98 ± 0.10
Linoleic acid (18:2n-6)	21.21 ± 1.98	13.27 ± 1.20 **	16.41 ± 1.55 *
Eicosadienoic acid (20:2n-6)	2.16 ± 1.37	0.92 ± 0.92	0.37 ± 0.37
Arachidonic acid (20:4n-6)	7.69 ± 0.73	4.47 ± 1.23 *	8.90 ± 1.35 +
**Triacylglycerol**			
Myristic acid (14:0)	1.23 ± 0.03	1.33 ± 0.08	1.39 ± 0.28
Palmitic acid (16:0)	21.80 ± 0.29	21.48 ± 0.63	21.02 ± 0.65
Stearic acid (18:0)	3.35 ± 0.15	5.27 ± 0.35 **	4.84 ± 1.68 *
Palmitoleic acid (16:1n-7)	2.50 ± 0.27	2.29 ± 0.34	2.44 ± 0.46
Oleic acid (18:1n-9)	24.37 ± 1.04	48.87 ± 1.35 **	47.09 ± 0.20 **
Vaccenic acid (18:1n-7)	1.98 ± 0.12	1.59 ± 0.12	1.73 ± 1.20
Linoleic acid (18:2n-6)	38.22 ± 1.36	16.59 ± 0.72 **	18.37 ± 0.16 **
α-linolenic acid (18:3n-3)	2.85 ± 0.16	0.67 ± 0.06 **	0.71 ± 0.20 **

Data are the mean ± SEM. Values significantly different from those obtained in the group of rats fed a chow diet (REF) are indicated by * (p < 0.05) and ** (p < 0.01). Data measured in the group of rats treated with ZD7114 (CAF-ZD) and significantly different from those in the group of rats fed a cafeteria diet (CAF) are shown by + (p < 0.05) and ++ (p < 0.05). Only main fatty acids are presented. CAF-ZD: rats fed a cafeteria diet and treated with ZD7114.

ZD7114 treatment did not change the proportions of any lipid classes measured in adipose TAG of CAF rats (Figure 2). However, in PL, the percentage of PUFA was significantly increased by 1.3 fold in CAF-ZD rats compared to CAF animals. This change was essentially due to a two-fold increase in the proportion of arachidonic acid measured in CAF-ZD rats (Table 3). A slight reduction in the percentage of myristic acid (1.2 fold decrease) was measured in CAF-ZD rats compared to CAF animals.

### Skeletal muscle lipids

#### Lipid content

PL, diacylglycerol (DAG) and TAG contents were measured in the gastrocnemius of REF animals and CAF rats with or without ZD7114 treatment (Table 4).

**Figure 3:**
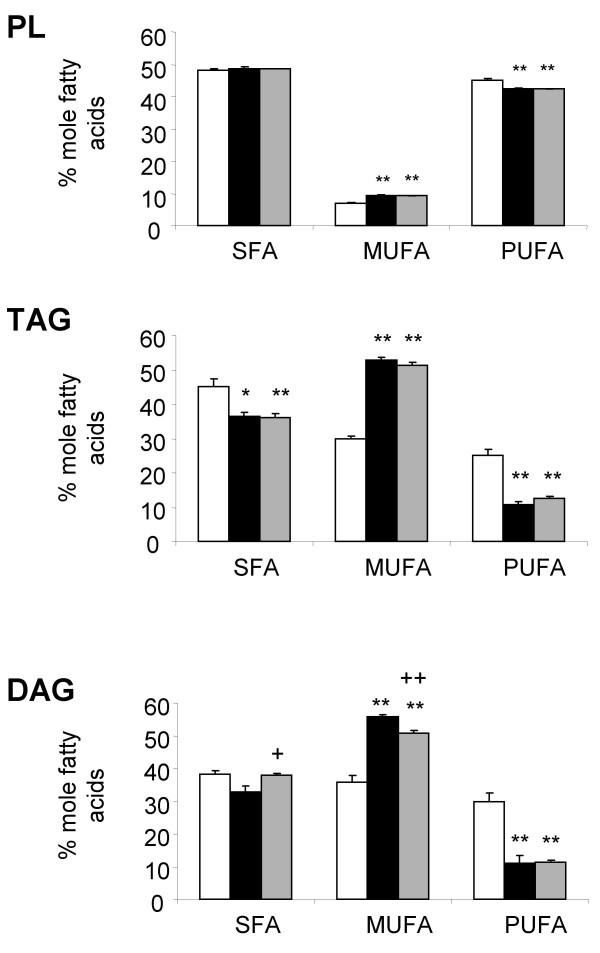
Proportion of the different lipid classes in muscle. Saturated (SFA), monounsaturated (MUFA) and polyunsaturated fatty acids (PUFA) were measured in muscle phospholipid (PL), triacylglycerol (TAG) and diacylglycerol (DAG) of rats fed a chow diet (empty bars), a cafeteria diet (black bars) alone or treated with ZD7114 (grey bars). Data are represented as mean ± SEM, and values significantly different to data measured in rats fed the chow or cafeteria diet are indicated by * (p < 0.05), ** (p < 0.01) or + (p < 0.05), ++ (p < 0.01), respectively.

The cafeteria diet clearly induced TAG and DAG accumulation in the gastrocnemius, as demonstrated by the respective 3.4 and 2.0 fold increases observed in CAF vs. REF rats (Table 4). Chronic treatment with ZD7114 did not significantly reduce the cafeteria diet induced-accumulation of TAG in muscle. In contrast, DAG accumulation was prevented as indicated by a 1.5 fold reduction of DAG levels in CAF-ZD when compared with CAF rats (Table 4). Values of intramyocellular TAG and DAG obtained in the present study (TAG: between 1 to 4 μmol/g fresh muscle; DAG: between 0.5 to 1 μmol/g fresh muscle) were similar to those previously described in rat skeletal muscles [31] (TAG: between 4 to 5 μmol/g fresh muscle; DAG: between 0.5 to 2.5 μmol/ g fresh muscle).

**Table 4:** Muscle lipid content

μmole/g tissue	**REF**	**CAF**	**CAF-ZD**
**Phospholipids**	60.31 ± 4.28	65.62 ± 1.26	70.80 ± 2.77
**Triacylglycerol**	5.40 ± 0.98	18.63 ± 3.24 **	15.65 ± 2.57 **
**Diacylglycerol**	2.07 ± 0.32	4.08 ± 0.24 **	2.66 ± 0.25 ++

Data are the mean ± SEM. Values significantly different from those obtained in the group of rats fed a chow diet (REF) are indicated by ** (p < 0.01). Data measured in the group of rats treated with ZD7114 (CAF-ZD) and significantly different from those in the group of rats fed a cafeteria diet (CAF) are shown by ++ (p < 0.05).

#### Fatty acid composition

Fatty acid profiles were determined in muscle PL, TAG and DAG. The gastrocnemius of CAF rats, compared with the REF group, presented an increase in the percentage of MUFA and a decrease in the proportion of PUFA in both TAG and DAG and to a lesser extent in PL (Figure 3). Variations in the percentage of oleic and linoleic acids were, respectively, responsible for the changes in the proportions of MUFA and PUFA in muscle TAG and DAG (Table 5). Modifications of the proportions of PUFA observed in muscle PL of CAF rats were due to slight decreases of both linoleic (1.4 fold increase) and docosahexaenoic (22:6 n-3) acids (1.3 fold increase), whereas the percentage of arachidonic acid (20:4 n-6) was increased by 1.2 fold.

**Table 5:** Composition of muscle lipids

% mole fatty acids	**REF**	**CAF**	**CAF-ZD**
**Phospholipids**			
Palmitic acid (16:0)	27.87 ± 0.50	26.17 ± 0.29	26.44 ± 0.57
Stearic acid (18:0)	20.21 ± 0.64	22.37 ± 0.54 *	21.64 ± 0.58
Oleic acid (18:1n-9)	6.84 ± 0.21	9.13 ± 0.48 **	9.12 ± 0.24 **
Linoleic acid (18:2n-6)	13.09 ± 0.65	8.98 ± 0.51**	9.02 ± 0.28 **
Arachidonic acid (20:4n-6)	11.64 ± 0.46	15.07 ± 0.53**	14.11 ± 0.22 **
Docosatetraenoic acid (22:4n-6)	ND	0.61 ± 0.04	0.51 ± 0.02
Adrenic acid (22:5n-3)	2.04 ± 0.07	2.44 ± 0.18	2.10 ± 0.09
Docosahexaenoic acid (22:6n-3)	18.30 ± 0.95	15.23 ± 0.52 *	16.09 ± 0.35
**Triacylglycerol**			
Myristic acid (14:0)	ND	1.66 ± 0.08	1.53 ± 0.06
Palmitic acid (16:0)	37.13 ± 2.06	25.26 ± 0.95 **	25.15 ± 0.81 **
Stearic acid (18:0)	7.94 ± 0.31	9.36 ± 0.45 *	9.38 ± 0.65
Palmitoleic acid (16:1n-7)	ND	1.51 ± 0.14	1.31 ± 0.13
Oleic acid (18:1n-9)	29.00 ± 0.94	51.36 ± 1.83 **	50.14 ± 0.93 **
Linoleic acid (18:2n-6)	25.00 ± 1.98	10.86 ± 0.83 **	12.47 ± 0.66 **
**Diacylglycerol**			
Myristic acid (14:0)	1.92 ± 0.09	1.75 ± 0.07	1.85 ± 0.05
Palmitic acid (16:0)	25.55 ± 0.86	22.42 ± 1.22	24.88 ± 0.62
Stearic acid (18:0)	6.71 ± 0.45	8.74 ± 0.63 *	9.71 ± 0.08 **
Palmitoleic acid (16:1n-7)	2.58 ± 0.20	1.59 ± 0.14 **	1.26 ± 0.07 **
Oleic acid (18:1n-9)	28.90 ± 1.24	52.88 ± 1.25 **	46.45 ± 1.61 ** ++
Nervonic acid (24:1n-9)	3.92 ± 1.93	1.24 ± 0.42	2.71 ± 0.88
Linoleic acid (18:2n-6)	26.96 ± 2.22	10.14 ± 2.04 **	8.05 ± 0.69 **
α-linolenic acid (18:3n-3)	1.12 ± 0.18	ND	ND
Stearidonic acid (18:4n-3)	ND	ND	1.90 ± 0.41
Arachidonic acid (20:4n-6)	0.78 ± 0.06	0.46 ± 0.06 **	0.26 ± 0.04 ** +

Data are the mean ± SEM. Values significantly different from those obtained in the group of rats fed a chow diet (REF) are indicated by * (p < 0.05) and ** (p < 0.01). Data measured in the group of rats treated with ZD7114 (CAF-ZD) and significantly different from those in the group of rats fed a cafeteria diet (CAF) are shown by + (p < 0.05) and ++ (p < 0.05). Fatty acids representing more than 1% in at least one group are presented.

The influence of ZD7114 on the cafeteria-induced modification of fatty acid composition in the three lipid species was evaluated by comparing CAF rats with CAF-ZD rats. While ZD7114 did not affect the fatty acid profile of either TAG or PL in muscle of CAF rats, it induced changes in the fatty acid composition of muscle DAG. Indeed, the proportion of SFA was slightly (1.2 fold increase), but significantly, elevated in DAG of CAF-ZD rats with an increase in the proportion of palmitic and stearic acids (Table 5). Furthermore, the proportion of MUFA in DAG of CAF-ZD rat muscles was decreased compared with DAG of CAF rats. This change was essentially attributed to a decrease in the proportion of oleic acid (Table 5). Stearidonic acid (18.4 n-3) was only detected in DAG accumulated in muscles of ZD-CAF rats. No differences in the proportion of PUFA between DAG stored in muscles of CAF-ZD or CAF rats were observed (Figure 3).

## Discussion

The present study demonstrates that feeding the cafeteria diet to rats not only promotes weight gain, hyperinsulinemia and hypertriglyceridemia, but also accumulation of lipids in skeletal muscle. Although TAG levels per gram of adipose tissue was not affected by the cafeteria diet, this dietary intervention strongly enhanced TAG accumulation in skeletal muscle, as previously described [4,5]. We show that this intramyocellular accumulation of TAG was associated with an increase in skeletal muscle DAG content. This finding is in agreement with the accumulation of DAG observed in human skeletal muscle cells incubated with saturated fatty acids *in vitro *[12]. DAG mass was also described to be increased in skeletal muscle biopsies obtained from normal volunteers in whom insulin resistance was produced by raising FFA levels during a lipid infusion [32]. However, to our knowledge, the present study demonstrates for the first time that a high fat diet, resembling the human Western diet, was able to increase DAG storage in skeletal muscle. Although both TAG and DAG accumulations in skeletal muscle were reported to be correlated with insulin resistance [4,5,33], DAG (a precursor of TAG synthesis) is proposed to directly impair insulin sensitivity by inactivating insulin receptor activity through activation of the protein kinase C [34].

The fatty acid composition of TAG stored in muscle was previously shown to be affected by dietary lipids [2]. Interestingly, we also find that the fatty acid composition of muscle DAG was modified by the cafeteria diet. Indeed, the increase in MUFA and the reduction of PUFA proportions, measured in both DAG and TAG, reflected the difference in lipid composition of the two diets (+ 19% MUFA and -28% PUFA in cafeteria diet vs. chow diet). It is noteworthy that qualitative changes in DAG were reported to affect the activity of DAG as a secondary messenger, since specificity of its fatty acyl moieties for the activation of protein kinase C has been described [35,36]. Although measurements of insulin sensitivity were not directly accessed in this study, our observation suggests that diet may regulate insulin response in muscle by modifying DAG composition.

Fatty acid compositions of muscle and adipose tissue PL were also modified by the cafeteria diet. More specifically, the proportion of PUFA in PL was decreased and MUFA was increased in both tissues. These differences were more pronounced in PL from adipose tissue than from gastrocnemius, indicating that membrane phospholipids in adipose tissue are more susceptible to variations in dietary composition than skeletal muscle. Interestingly, reductions in the proportion of PUFA in PL from myocytes [7] and adipocytes [8] were reported to impair insulin action.

As previously reported with Trecadrine, a selective β3-AR agonist [22], chronic administration of ZD7114 prevented the excess weight gain and fat accumulation induced by a cafeteria diet. This present report demonstrates that ZD7114 treatment reduced the hyperinsulinemia elicited by the cafeteria diet (by 2.3 fold), thereby suggesting an improvement of insulin sensitivity in the treated rats. This observation was associated with a significant reduction in the accumulation of skeletal muscle DAG, and a slight, non-significant decrease in TAG. It is tempting to suggest that the decrease in muscle DAG accumulation induced by the chronic administration of a β3-AR agonist could participate in the prevention of hyperinsulinemia. β3-AR agonist treatment significantly modified the fatty acid composition of DAG, causing a 1.2 fold reduction in the proportion of oleic acid. As mentioned before, these modifications in DAG composition could directly regulate insulin action in muscles. Since the presence of a β3-AR in skeletal muscle is still a subject of debate, it is difficult to elucidate the mechanism(s) by which the β3-AR agonist lowers DAG content in muscle and modifies its fatty acid composition.

Although the content and the fatty acid composition of TAG stored in adipose tissue was not affected by β3-AR agonist administration, the fatty acid profile of adipose PL was modified by the treatment. The β3-AR agonist was able to restore the proportion of PUFA to a level similar to the REF rats by increasing the percentage of arachidonic acid by 2 fold. As mentioned above, a greater percentage of PUFA in PL has been reported to enhance insulin sensitivity in adipose tissue. The changes in PL composition induced by the β3-AR agonist may be associated with the previously reported improved insulin sensitivity in adipose tissue [8], and the decrease of hyperinsulinemia measured in the present study.

## Conclusions

Whilst the effectiveness of β3-AR agonists is limited in humans, understanding the metabolic changes affected in rodents will provide insights into mechanisms, underlying insulin responsiveness in humans. The present study demonstrates that β3-AR agonist treatment not only limits the hyperinsulinemia induced by a cafeteria diet, but also partially prevents the associated alterations in adipose and muscle lipid composition. Particularly, activation of the β3-AR limits the intramyocellular DAG accumulation and the decrease in the proportion of PUFA in adipose tissue PL. This combined action may contribute to the beneficial effect of β3-AR agonists on insulin sensitivity.

## List of abbreviations

*CAF rats*: Rats fed with a cafeteria diet; *DAG*: Diacylglycerol; *MUFA*: Monounsaturated fatty acid; *PKC*: Protein kinase C; *PUFA*: Polyunsaturated fatty acid; *REF rats*: Rats fed with a chow diet; *SFA*: saturated fatty acid. *TAG*: Triacylglycerol; *ZD-CAF rats*: Rats fed with a cafeteria diet and treated with the β3-AR agonist ZD7114.
